# Seed-Mediated, Shape-Controlled Synthesis Methods for Platinum-Based Electrocatalysts for the Oxygen Reduction Reaction—A Mini Review

**DOI:** 10.3389/fchem.2022.865214

**Published:** 2022-03-04

**Authors:** Daisy E. Gray, Tasnim Munshi, Ian J. Scowen, Dan J. L. Brett, Guanjie He

**Affiliations:** ^1^ Joseph Banks Laboratories, School of Chemistry, University of Lincoln, Lincoln, United Kingdom; ^2^ Department of Chemical Engineering, University College London, London, United Kingdom

**Keywords:** platinum, electrocatalysts, hydrogen fuel cell, oxygen reduction reaction, shapecontrolled, seed-mediated

## Abstract

Overcoming the slow oxygen reduction reaction (ORR) kinetics at the cathode of the hydrogen fuel cells requires the use of electrocatalysts containing expensive and scare platinum to achieve reasonable performance, hampering widespread use of the technology due to high material costs and sustainability issues. One option available to tackle this issue is to use new designs to create nanomaterials which achieve excellent electrocatalytic performances and long-lasting stabilities whilst using less platinum than is currently required. Reliably producing nanomaterials with predictable activities and stabilities using simple, safe, and scalable methods is an important research topic to the advancement of fuel cell technologies. The oxygen reduction reaction occurs at the surface of electrocatalytic materials, and since nanomaterial structures exhibit different catalytic activities, their shapes have a strong relationship to the final performance. Seed-mediated synthesis can be used to control the shape of materials with the aim of obtaining products with the most desirable surface properties for the ORR. This review summarized the current advancement of the synthesis of platinum-based ORR and provided the insights for the future development of this field.

## Introduction

To achieve excellent performance and reduce the amount of platinum required in electrocatalytic materials for the oxygen reduction reaction, design strategies aim to optimise a materials interactions with oxygen and intermediate species. This is achieved by altering electronic structure through alloying and inducing strain, and by exposing a sufficient amount of preferential active sites though controlling size and shape. In this review the use of seed-mediated growth as a synthesis technique to produce nanocrystals with tailored shapes as a means of improving catalytic performance is discussed.

**FIGURE 1:**
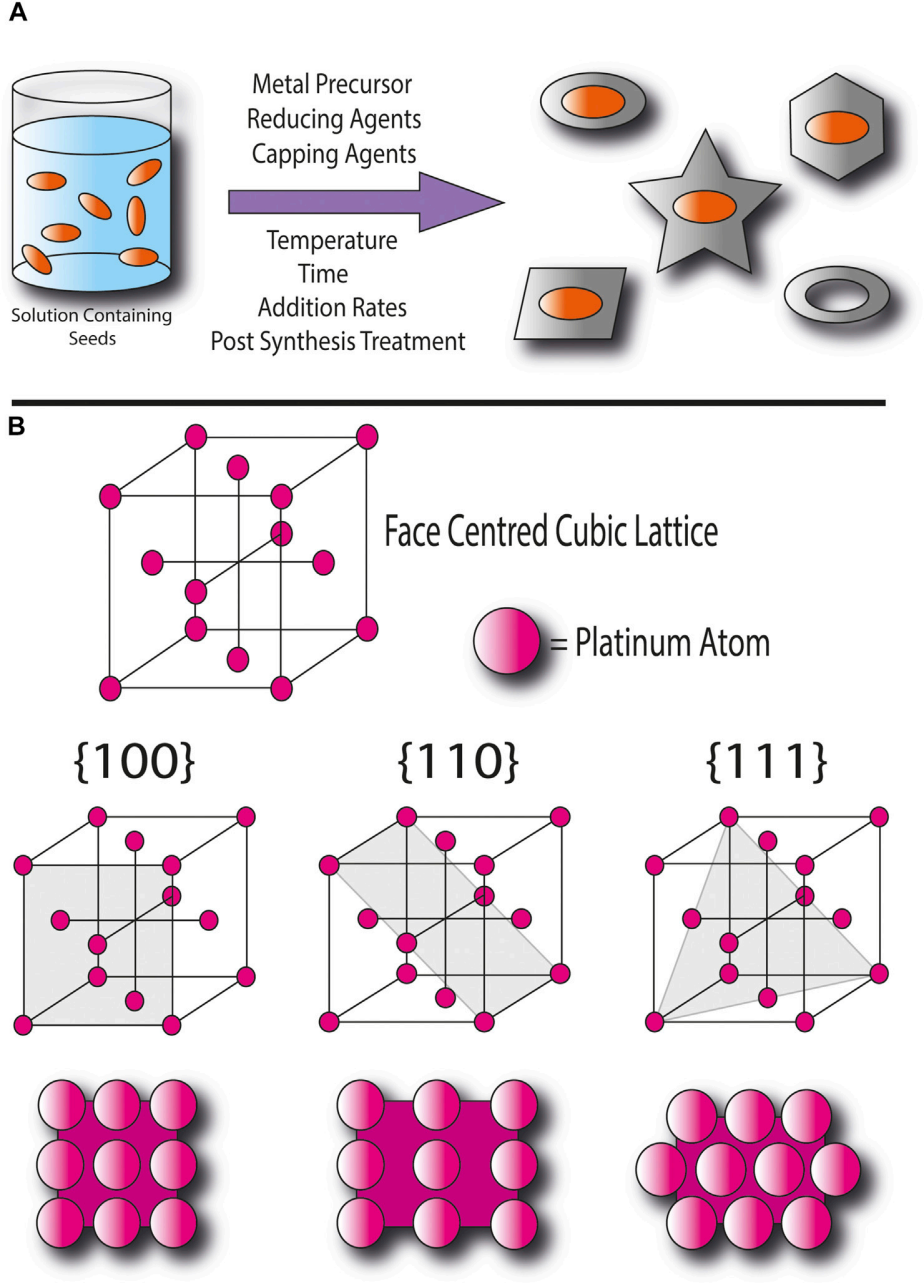
**(A)** Illustration of the general procedure of seed-mediated synthesis to produce different nanocrystal shapes, **(B)** Illustration of platinum’s face centred cubic lattice and its {100}, {110}, and {111} facets.

The general method of seed-mediated synthesis involves using nanoparticle seeds as sites for homogenous nucleation, during which atoms deposit onto the seed surface and grow into nanocrystals (Xia et al., 2016). The direction of growth in a nanocrystal is determined by the energies of its different facets, with the presence of more stable ones being favored in the product. The control of the kinetics or thermodynamics of the reaction can be used to promote or inhibit the growth of certain crystal facets and this is achieved by optimizing reaction conditions such as choosing reducing and stabilizing agents, controlling the concentrations of precursors and reactants, temperatures, reaction times, and rates of feeding (Figure 1A) (Poerwoprajitno et al., 2019).

T. Wang et al. provides a detailed description of the mechanism of the oxygen reduction reaction (the ORR) at precious metal surfaces and the effect of the strength of the metal’s interaction with oxygen species on activity. Since the ORR involves the adsorption of O_2_ onto active sites at the catalyst surface, a sufficient number of sites with appropriate strengths of adsorption of oxygen are required to achieve reasonable rates of reaction (Wang et al., 2021). The catalytic activities and selectivity of a nanostructured materials facets could differ due to different types of adsorption sites available and the exposure of the second surface layer. Figure 1B depicts the face-centered cubic lattice of the platinum, and its three most commonly observed and studied facets ({100}, {110}, and {111}). In {100} and {111} facets, the adsorption sites could be at single atoms, at the bridges between two atoms, or in between four and three atoms respectively, and the facets have relatively atomically smooth surfaces. In the {110} facet, adsorption at bridges between atoms could occur at either the atoms next to one another within rows or at longer bridges between rows, and at gaps between rows where the second surface layer is slightly exposed. The surface is also rougher than {100} the and {111} facets. Single-crystal and pure Pt facets have been reported to have activities in the order of {100} < {111} < {110}, whilst for Pt and metal alloy facets, the activities follow the order of {100} < {110} < {111} (Yang et al., 2018). Whereas low-index facets are densely packed, high-index facets contain structural features such as terraces and steps and are higher in formation energy due to defects and the lower coordination numbers of their atoms. Therefore, higher-index facets have been found to demonstrate better catalytic activities but are also less stable and as a result a nanocrystal with high-index facets can be more difficult to synthesize (McCrum et al., 2017). The face-centered cubic lattice of the platinum can take several different crystalline forms, for example single crystals, twinned where the structure contains two crystals joined together, or multiply twinned where more than two joined crystals make the entire structures. This leads to a diverse range of shapes available, each enclosed by different ratios of facets.

The energies of nanocrystal facets can also change during the use as electrocatalytic materials due to species such as electrolyte anions adsorbing onto them, and changes in the potential during fuel cell operation. As a result of these factors, restructuring of nanocrystals is observed throughout their use. For example, Density functional theory (DFT) calculations have shown that at low and high potentials, Pt(100) and Pt(110) surfaces become more stable than Pt(111) due to the influence of adsorbed hydrogen and hydroxide, leading to their enhanced growth (Wang et al., 2020). The structural transformation during operation can affect the durability of the electrocatalyst, therefore producing materials with properties that result in improved stabilities is also an essential requirement in improving the affordability and sustainability of fuel cell technologies (Liu et al., 2018).

As a wet-chemical method, seed-mediated synthesis is a simple technique that does not require specialist equipment, reaction conditions are not harsh and are easily controllable, the reaction progression is easily observable, and it can be scaled to large manufacture. It is also a versatile method that can produce a wide range of nanostructured materials, as demonstrated by the following examples.

### Seed-Mediated Morphology

The selection of seeds is an important consideration when synthesizing a monodispersed product. The preparation of the seeds and their purity is an important factor in successful nanoparticle synthesis. For example, smaller seeds have been found to fluctuate in structures whereas larger seeds were found to be more stable and therefore obtainable in higher yields (WenXin et al., 2012). Seed-mediated synthesis aims at reproducing the seed morphology in the nanomaterials or transforming it through growth. The interaction between the seed and shell are also important, with properties such as electronegativities and atomic radii expected to affect the successful deposition of one material on another. For these the selection of both seed material and seed size are important considerations.

Li et al. synthesized Pd@Pt tetrapods and found that the small lattice mismatch between Pd and Pt as well as controlling deposition rate and synthesis temperature allowed for the retention of the seed morphology. An ultrathin (six atoms) layer of platinum was deposited on palladium tertrapod seeds, which were then removed *via* acidic etching leaving behind hollow platinum tetrapods (Pt HTPs). The product was found to retain structural features of the palladium seeds which are advantageous for the ORR, including a highly porous structure enclosed by {111} facets, and a concave topology that suggests the presence of steps at the surface. Injecting the Pt precursor slowly and keeping the reaction temperature sufficiently high can ensure that the Pt atoms were able to move over the surface of the seed and deposit uniformly instead of forming islands. The Pt HTPs were found to exhibit a higher ESCA than Pt/C (33.43 and 14.18 m^2^ g^−1^, respectively) and displayed better stability than Pt/C (E_1/2_ degradation of 3 mV after 1,000 cycles of accelerated durability test for Pt HTP compared with 21 mV degradation under the same conditions for Pt/C), demonstrating a structure that remains stable throughout the electrochemical application (Li et al., 2021a).

**FIGURE 2:**
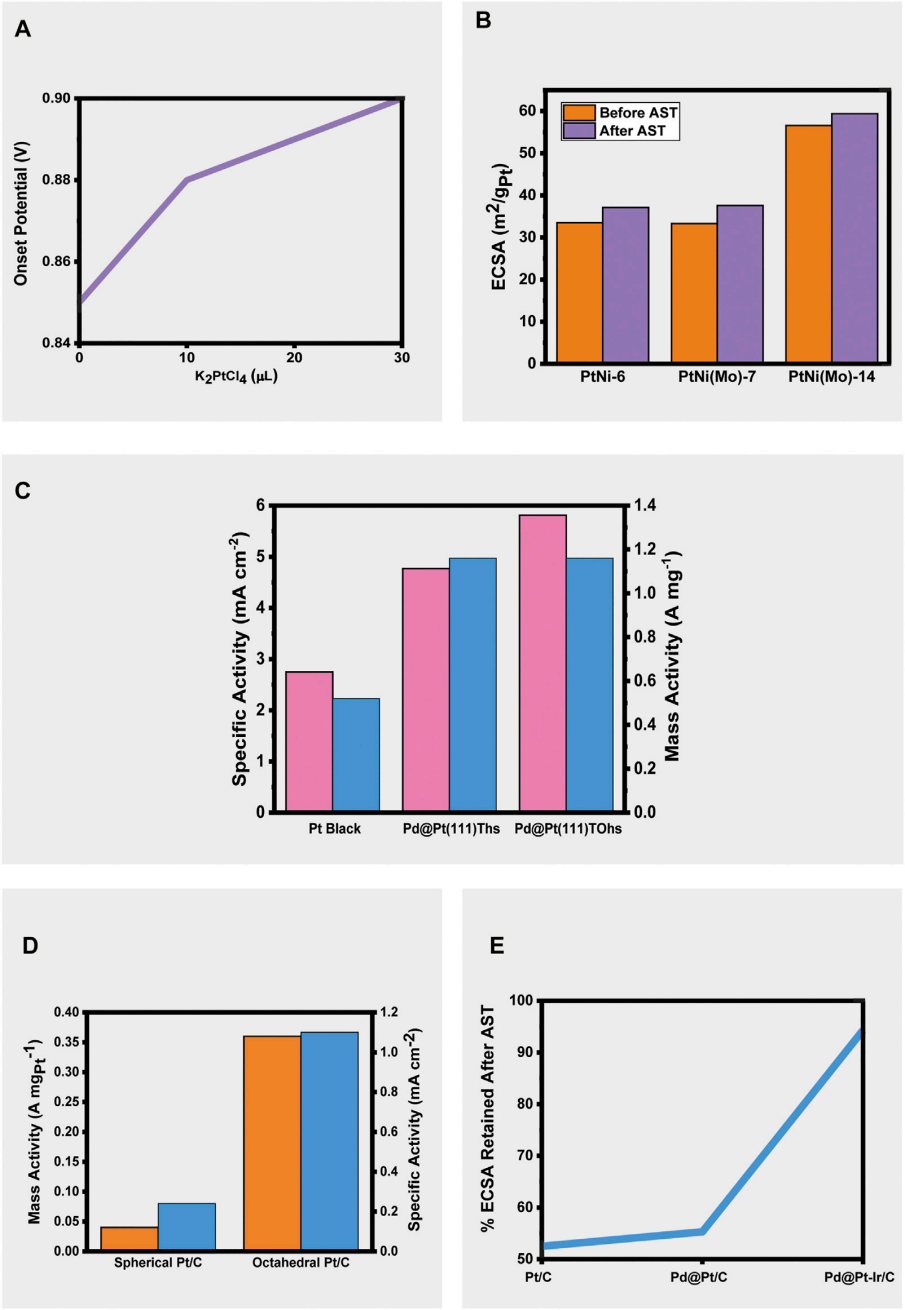
**(A)** Change in onset potential of Au@Pt CCB NCs with increasing platinum precursor amount (10), **(B)** Change in ECSAs of PtNi-6, PtNi(Mo)-7, and PtNi(Mo)-14 after 10,800 cycles of accelerated stress testing (17), **(C)** Comparison of specific and mass activities of Pt Black, Pd@Pd(111)Ths, and Pd@Pt(111)TOhs (13), **(D)** Comparison of mass and specific activities of spherical and octahedral Pt/C (16), **(E)** Percent of ECSA retained after accelerated stress testing for commercial Pt/C, Pd@Pt icosahedra/C and Pd@Pt-Ir Icosahedra/C (18).

At high concentrations of Pt, it is found that its deposition onto Au shells results in the morphology of the Au nanocubes (NCs) being lost as Pt grows into islands instead of diffusing into an even coverage. This can be explained due to a lattice mismatch between Pt and Au and the strong Pt-Pt bonds. Roy et al. (2021) achieved the smooth coverage of Pt whilst maintaining the seed morphology through the addition of Ag^+^ ions within an optimum range. Ag^+^ ions deposit onto the Au surface through under potential deposition and facilitate the deposition of Pt through either direction to preferential sites, or by galvanic replacement. 0, 10, 20, and 30 μL of AgNO_3_ were used, with 0, 20, and 30 μL resulting in no substantial formation of a Pt layer. The lack of successful deposition at higher amounts of Ag^+^ in the growth solution was explained due to the fast regeneration of the Ag layer on the Au shells before Pt can nucleate and deposit. Upon varying the amount of Pt precursors, it was found that the synthesized Au@Pt CCB NCs with high Pt loading displayed better activities for the ORR with higher onset potentials (Figure 2A), furthermore, the highest Pt-loading materials displayed better stability than commercial Pt/C after 5,000 cycles of accelerated stress test (shifts of 28 and 39 mV respectively). (Roy et al., 2021).

### Kinetic and Thermodynamic Control

Thermodynamics control which atomic positions and arrangements are most stable and therefore have a strong influence over which facets of a nanocrystal grow, however kinetics can also be used to manipulate the final shape. Kinetics favor the formation of nanostructures with defined shapes and ones of a larger size, whereas thermodynamics favor smaller and more spherical shapes. The kinetics of the reaction and the ultimate thermodynamic stability of the products are not separate properties, and both must be considered during seed-mediated nanocrystal growth (Gilroy et al., 2016).

The successful synthesis of ultrathin nanorings of PdPtCu with high-index facets and high aspect-ratios was achieved by Li et al. (2021a). These structures are usually difficult to synthesize due to their thermodynamic instability and metals predisposition to grow isotropically, but careful design of the synthesis method such as choice of reagents and order of reaction was used to overcome these difficulties. Pd-rich ultrathin nanosheets were used as seeds on which the reduced Pt diffused into, followed by the addition of Cu and oxidative etching of the sheets into rings by Br^−^/O_2_ pairs. It was found that adding all three metal precursors at once resulted in irregular mixtures of products, therefore controlling the order of reaction was necessary to achieve the desired nanostructure. The choice of reductant [W(CO)_6_] resulted in the inhibition of growth at {111} facets by CO capping, and the choice of structure-directing reagent (NH_4_Br) was essential in the formation of the ring structures. Pd_39_Pt_33_Cu_28_/C was found to have a substantially larger ECSA than Pt/C (92.2 m^2^ g^−1^_PGM_ and 62.8 m^2^ g^−1^_PGM_ respectively), and displayed better stability than Pt/C under accelerated durability testing (86% of activity retained compared to 37.1%, respectively). This superior performance was attributed to the optimization effect of the high-index facets and alloying with Cu on the oxygen adsorption energy (Li et al., 2021b).

Thermodynamically favored sub-10 nm Pd@Pt(111) tetrahedrons were synthesized by Su et al. by injecting a Pt precursor into a growth mixture containing sub-5 nm Pd tetrahedral seeds. It was found that the volume of precursor injected into the growth mixture affected the product obtained and that when the platinum shell exceeded five atomic layers in thickness, the tetrahedrons transformed into truncated octahedron shapes. The production of these thermodynamically favored products suggested that deposition occurred faster than diffusion. Had the opposite been true, the product would be expected to grow in a kinetic-controlled manner. The ORR activity was found to be superior for both the truncated octahedral structures (TOhs) and the original tetrahedral structures (Ths) when compared to Pt Black. Pd@Pt(111) TOhs had a higher ECSA (28.1 m^2^ g^−1^) compared to Pd@Pt(111) Ths (24.4 m^2^ g^−1^) and Pt Black (18.9 m^2^ g^−1^). Pd@Pt(111) TOhs had the highest mass and specific activities (1.632 A mg^−1^ and 5.809 mA cm^−2^, respectively), followed by Pd@Pt(111) Ths (1.164 A mg^−1^ and 4.770 mA cm^−2^, respectively). Pt black exhibited the lowest mass and specific activities of all three materials at 0.520 A mg^−1^ and 2.750 mA cm^−2^, respectively (Figure 2C). The truncated octahedral structure also displayed the best stability with a change in E_1/2_ of 18 mV over 30,000 cycles of accelerated stress testing compared to Pd@Pt(111) Ths (24 mV) and Pt Black (48 mV) (Su et al., 2021).

### Directing Shape Chemically

Chemicals that stabilize certain facets can be included in the synthesis procedure to control the shape of the product. Stabilized facets will grow at a slower rate and are therefore more likely to be present in the final product. Examples of commonly used shape directing agents include inorganic salts such as bromide and iodide salts, ligands such as CTAB and oleic acid, and polymers such as PVP (Leong et al., 2013).

The shape directing effect of using three different ternary mixtures of hydrophobic surfactants in the synthesis of core-shell Ni-Pt nanoparticles was investigated by Leteba et al. To mixtures of oleyamine (OAm) and octadecylamine (ODA) surfactants, [oleic acid (OLEA), dioctylamine (DOA), and trioxtylamine (TOA)] were added individually. Whereas the use of OAm and ODA are expected to result in the promotion of {110}-crystal facets when forming PtNi nanocrystals, the third surfactants encouraged different shapes, and therefore different electrochemical activities of the products. The addition of OLEA resulted in a major product of cuboidal and tetrahexahedral structures, whereas the addition of TOA and DOA resulted in major products which were larger in size and also contained rhombic dodecahedral and elongated polyhedral particles. The specific activities were found to outperform commercial Pt/C in the order of TOA > OLEA > DOA > Pt/C, (0.51, 0.39, 0.37, 0.057 mA cm_Pt_^−2^, respectively). Mass activities were also superior in the order of OLEA > DOA > TOA > Pt/C, (0.36, 0.31, 0.28, 0.093 A mg_Pt_^−1^ respectively). This was credited to a very thin layer of Pt, advantageous interactions between core and shell, and the complex surfaces of the products. After accelerated stress testing, all three products were found to lack stability, losing significant percentages of their specific and mass activities. It was noted that long-term stability needs further development (Leteba et al., 2020).

Conversely, the intentional omission of shape directing reagents such as surfactants was employed by Hornberger et al. to produce sub-10 nm Pt octahedral nanoparticles with majority {111} facets. This green synthetic method bypassed the challenges posed by the use of shape directing reagents, such as difficulties in producing very small sizes of nanoparticles (<10 nm), unwanted alterations to the catalytic behavior of the material, and difficulty in purification. The quality and purity of the Pt seeds were crucial to successful synthesis since monocrystallinity is an important factor when producing a reliable shape-controlled product. The use of sodium citrate as a shape directing agent required control of temperature as well as the rate of heating, and exposure to oxygen. Without these controls, a different shape product was obtained. The 13 wt% oh-Pt/C (octahedral) vastly outperformed 47.6 wt% s-Pt/C (spherical) reported in the literature, with mass activities of 0.36 and 0.04 A mg_Pt_^−1^, respectively, and specific activities of 1.10 and 0.24 mA cm^−2^, respectively (Figure 2D). The product showed good stability by retaining 86 and 85% of its mass and specific activities after 10,000 cycles of accelerated durability testing (Hornberger et al., 2021).

### Seed-Mediated Synthesis for Improved Stability

It is known that there is a discrepancy between the results of nanomaterial performance and stability obtained in the laboratory and those observed in real-world application. One consideration to be made in electrocatalyst design is that in real-world use within fuel cells, shape-controlled Pt-based catalysts suffer from a lack of durability. Seed-mediated synthesis has been used to produce materials with both improved stabilities and catalytic activities when compared to equivalent alternatives.

Polani et al. used a one-pot synthesis method to prepare nanocrystals with different edge lengths. The edge lengths were found to be tuneable by controlling the amount of Ni precursor added to the reaction, with an increased amount resulting in a larger nanocrystal. The composition and distribution of atoms was found to change with size. In the smaller crystals Pt and Ni were found to be evenly distributed, whereas in the larger crystals Pt and Ni were found to be more segregated with Pt-rich edges and Ni at the facets. The Ni in less evenly distributed bimetallic PtNi nanocrystals is known to leach resulting in the loss of the catalytically active {111} facets. A trend was observed of higher specific activities, ECSA’s, and therefore mass activities with increasing crystal size. When compared to undoped PtNi-6, it was found that the addition of Mo improved performance and durability. PtNi-6 had an ECSA of 33.5 m^2^ g_Pt_^−1^, a specific activity of 0.5 mA cm^−2^ and a mass activity of 0.32 A mg_Pt_^−1^, whereas PtNi(Mo)-14 had an ECSA of 56.6 m^2^ g_PT_^−1^, a specific activity of 5.84 mA cm^−2^, and a mass activity of 1.95 A mg_Pt_^−1^. After accelerated stress testing the undoped PtNi-6 specific and mass activities improved but still did not reach the values of PtNi(Mo)-14 (Figure 2B) (Polani et al., 2021)

Zhu et al. found that alloying Pt with Ir to produce Pd@Pt-Ir core-shell nanocrystals resulted in better performance and stability compared to Pd@Pt/C materials. Pt and Ir were simultaneously deposited in thin layers over cubic, octahedral, and icosahedral Pd seeds with {100} and {111} facets. A slow injection of precursors and a high temperature were maintained to obtain nanocrystals with a smooth shell. At low temperatures deposition occurred faster than diffusion which ultimately affected the shape of the nanocrystals. At a fast injection rate or with the omission of the reductant, KBr control over the nanocrystal shape was lost. The ESCA of the icosahedral/C nanocrystals was the highest of those reported and outperformed commercial Pt/C (121.7 m^2^ g^−1^_Pt+Ir_ compared to 59.4 m^2^ g^−1^_Pt_ respectively). This material also only lost 5.7% of its ECSA after accelerated stress testing compared to the 47.5% loss of commercial Pt/C catalysts (Figure 2E). The superior performance of the icosahedral/C product was attributed to its larger amount of {111} facets and twin boundaries. Compared to Pd@Pt/C materials with no Ir, these materials exhibited up to 2.9 times better mass activities. The stability of the Pd@Pt-Ir core-shell alloys was believed to be due to the inclusion of Ir- since whereas Pd suffers from leaching Ir remains stable in acidic conditions. It was noted that in the future work a less scarce material than Pd should be pursued as a core material (Zhu et al., 2020).

## Conclusion

This review summarized several representative examples of seed-mediated and shape-controlled synthesis of platinum-based nano-electrocatalysts reported recently. From these examples, it can be seen that this synthesis method is capable of producing a host of interesting materials that exhibit promising electrocatalytic performances due to the promotion of specific facets. When assessed using accelerated stress testing, this synthesis method has been shown to produce electrocatalysts that are more durable than comparable materials.

There are challenges to be addressed in the future of electrocatalytic nanomaterial development. In the context of shape-controlled nanomaterials, these challenges include gaining a better understanding of the growth mechanisms of specific crystal facets and how to promote them more reliably, the changes that occur to surface facets whilst taking part in electrochemical reactions, and how to control these changes or use them to promote activity and stability. The synthesis methods need to be scale-up from the lab to large-scale manufacture, which will require the continued development of reliable and safe methods with low energy and cost requirements. When adopting seed-mediated synthesis methods the use of expensive and scarce metals as seeds should also be addressed, for example by replacing these materials with transition metals. When using stabilizing agents, more precise knowledge of their influence on specific facet growth, and better procedures for their removal is also necessary. As with all materials design, the lengthy and laborious method of synthesizing and then testing a large number of electrocatalytic materials can be significantly reduced in the future with the development of computer modelling to predict activities and stabilities.
